# To take or not to take?

**DOI:** 10.3325/cmj.2015.56.513

**Published:** 2015-12

**Authors:** Hans Joachim Seitz

**Affiliations:** 1 Southeast-Europe Cooperation, Universitätsklinikum Hamburg-Eppendorf, Hamburg, Germany; 2 Corresponding member of the Croatian Academy of Science and Arts, Zagreb, Croatia; 3 Member of the Executive Committee of the Inter-University Center Dubrovnik, Dubrovnik, Croatia; 4 Member of the International Scientific Board of Ruđer Bošković Institute, Zagreb, Croatia

To take, or not to take? That is the question on how to develop science in Croatia. 

One of the tasks of the *Croatian Medical Journal* (CMJ) is the promotion of Croatian science. Although it started as a regional journal, the *CMJ* developed through the years and began to play a global role, helping Croatian scientists to present their results to the international biomedical community. The *CMJ* acts as a window to the world for Croatian medical professionals, and the articles published in the *CMJ* serve as a benchmark of quality in the Croatian medical science. Still, in order to be successful, Croatian scientists have to overcome many obstacles, the major one being the lack of funding.

Recently (in 2013), Croatia joined the European Union (EU), and although some aspects of the EU’s functioning have recently come under considerable criticism, Croatia is in a very favorable position. The citizens of Croatia should be aware of the fact that this country was awarded €11 billion from cohesion funds at the occasion of becoming an EU member. A considerable part, ie €3 billion, are already available and should be spent by the end of 2016. The money is not explicitly intended only for building roads and railways but cca. €500 million is intended for development of infrastructure, eg, for universities and hospitals. In the early 2014, members of the Croatian Government had a meeting with the top administrators in the German Government in Berlin. The purpose was to congratulate Croatia for obtaining this tremendous financial support and to offer it strategic and administrative help to spend it in the best possible way. In small group meetings of experts from Croatia and Germany, numerous ideas were presented, and the enthusiasm on both parts was overwhelming. Now, as I visited Croatia nearly two years later and talked with members of the medical community and scientists, I could get no answers as to how the money was spent. Instead I was faced with all-pervading melancholia. The process was not transparent, nor were the sources of financing visible to scientists in Croatia!

A recent World Bank Report describes what all Croatian families know: the educated, bright, often multilingual young people run away. The brain drain should be counteracted with brain gain, and the money received should be directed to the local young and perspective professional groups, which flourish regardless to the circumstances. Still, it seems that the democratic dialogue and the tools of public communication have not been used to the expected extent. Where is the voice of the Medical Chamber/Association, the Croatian Academy of Science and Art, the Croatian Medical Academy, the Rectors of Universities, the Deans of Faculties, of those most responsible members of the Croatian Civil Society, who are of course aware of the consequences of such a situation? Sadness inside the families, sadness about losing the gifted students, nurses, technicians, medical engineers in clinics and institutes, sadness about a lonely future in a country that slowly gets older and older. In 10 or 15 years to whom will this Civil Society pass on the keys of (museum-like) institutes or clinics with outdated standards?

My major argument here is that the money should be awarded to the young and promising professionals who need to pursue their careers in Croatia. The transparency of the process and the visibility of the achievements is crucial for the nation to feel the strength of its own progress.

What to do in this really grotesque situation? We have nearly unlimited financial resources on the one hand and young educated people without access to these resources on the other. Here are the suggestions in the form of an Action Plan:

• Organize task forces (with international participation) for carrying out realistic projects.

• Make substantial investment into a few major university hospitals and research institutes in order to ensure their competitiveness at the European and global level. The institutions should also focus on a few selected projects and abandon the others. This would ensure fair distribution of excellent specialized medicine throughout Croatia.

• Bring together promising research groups in a few Campus Research Centers to allow for rational resource use and interdisciplinarity.

• Promote these Centers of Excellence in Medicine, give them a clear structure, and demand success.

• Provide the institutions with autonomy (finances, employees, investments), but also a strong management under control by task forces, and use reliable indicators to measure their success, while the institutions themselves are to provide the public with a realistic master plan.

• Immediately establish a generous Croatian PhD program (scholarships and money for running expenses) with international links.

• Immediately establish a generous Croatian postdoc program (success dependent bonus system and money for running expenses) with international links.

Now it is time for Croatians to make a move. It is up to them to choose an action plan, similar or different from the one presented here. Whatever plan they choose they must make sure that it addresses the burning issues that Croatia faces today – brain drain, brain gain, and the impact of the invested money on the young and promising population of Croatia.

